# Rapid assessment of visual impairment (RAVI) in marine fishing communities in South India - study protocol and main findings

**DOI:** 10.1186/1471-2415-11-26

**Published:** 2011-09-19

**Authors:** Srinivas Marmamula, Sreenivas R Madala, Gullapalli N Rao

**Affiliations:** 1 International Centre for Advancement of Rural Eye care, L V Prasad Eye Institute, Hyderabad, India; 2 Bausch & Lomb School of Optometry, L V Prasad Eye Institute, Hyderabad, India; 3 Rajiv Gandhi Institute of Medical Sciences, Ongole, Andhra Pradesh, India

**Keywords:** cataract, fishing communities, rapid assessment, India, visual impairment

## Abstract

**Background:**

Reliable data are a pre-requisite for planning eye care services. Though conventional cross sectional studies provide reliable information, they are resource intensive. A novel rapid assessment method was used to investigate the prevalence and causes of visual impairment and presbyopia in subjects aged 40 years and older. This paper describes the detailed methodology and study procedures of Rapid Assessment of Visual Impairment (RAVI) project.

**Methods:**

A population-based cross-sectional study was conducted using cluster random sampling in the coastal region of Prakasam district of Andhra Pradesh in India, predominantly inhabited by fishing communities. Unaided, aided and pinhole visual acuity (VA) was assessed using a Snellen chart at a distance of 6 meters. The VA was re-assessed using a pinhole, if VA was < 6/12 in either eye. Near vision was assessed using N notation chart binocularly. Visual impairment was defined as presenting VA < 6/18 in the better eye. Presbyopia is defined as binocular near vision worse than N8 in subjects with binocular distance VA of 6/18 or better.

**Results:**

The data collection was completed in <12 weeks using two teams each consisting of one paramedical ophthalmic personnel and two community eye health workers. The prevalence of visual impairment was 30% (95% CI, 27.6-32.2). This included 111 (7.1%; 95% CI, 5.8-8.4) individuals with blindness. Cataract was the leading cause of visual impairment followed by uncorrected refractive errors. The prevalence of blindness according to WHO definition (presenting VA < 3/60 in the better eye) was 2.7% (95% CI, 1.9-3.5).

**Conclusion:**

There is a high prevalence of visual impairment in marine fishing communities in Prakasam district in India. The data from this rapid assessment survey can now be used as a baseline to start eye care services in this region. The rapid assessment methodology (RAVI) reported in this paper is robust, quick and has the potential to be replicated in other areas.

## Background

Visual impairment is a global public health challenge. Cataract and uncorrected refractive errors constitute the leading causes of visual impairment in most parts of the developing world, including India [1]. Recently, a landmark paper has shown that over 410 million people have uncorrected presbyopia [2]. Most of the cases of refractive errors and presbyopia can be easily corrected with spectacles, and cataract can be addressed by surgery. Cataract surgery is a cost-effective intervention in eye care [3]. Despite such simple remedies, uncorrected refractive errors cause 16% of the blindness and 46% of the visual impairment across all age groups in the Indian state of Andhra Pradesh [4,5].

Although population-based cross sectional studies like APEDS provide reliable information for setting priorities and starting up the services, they are prohibitively expensive, time consuming and require expertise in epidemiology. Hence, a methodology is required to estimate the burden of the problem and also to provide baseline data to plan the services using limited resources. Several rapid assessment methods are used for this purpose [6-11]. Rapid Assessment of Cataract Surgical Services (RACSS) is one of the earliest of rapid assessment methods used in eye care [12]. In RACSS the main focus is prevalence of cataract and cataract surgical services. The Rapid Assessment of Avoidable Blindness (RAAB) is a recent rapid assessment method that is more comprehensive and includes all the causes of visual impairment [7,13].

Though RAAB provides comprehensive information on prevalence and causes of visual impairment, the information on uncorrected refractive errors is limited to prevalence only. It does not provide information on spectacle use and coverage, both of which are important indicators for assessing the penetration of eye care services in the region. 'Willingness to pay' is an important indicator that is essential to planning the pricing system for eye care services. The RAAB also does not provide information on uncorrected presbyopia, which contributes to a significant proportion of near visual impairment globally [2].

We used a novel rapid assessment methodology, titled 'Rapid Assessment of Visual Impairment (RAVI)' in marine fishing communities in Prakasam district in Andhra Pradesh to estimate the prevalence and common causes of visual impairment, prevalence of presbyopia, spectacle coverage, cataract surgical coverage, barriers for uptake of eye care services and 'willingness to pay' for cataract surgery and spectacles for uncorrected refractive errors and presbyopia. This paper describes the detailed methodology of the RAVI project and its main findings.

The fishing communities were selected because they are marginalized population and have distinct life style compared to other communities in the region. The literacy rates are low compared to the overall literacy of the district. Till date, there are no data on visual impairment from these communities. There was also a plan to provide eye care services at no cost for those who have visual impairment.

## Methods

### Sample size selection

The sample size for the study was calculated using RAAB software [7]. The parameters used for this were; the assumed 8% prevalence of visual impairment (presenting visual impairment < 6/18) among those aged 40 years and older, relative precision of 0.5% (± 20% of the prevalence), 95% confidence intervals, assumed response rate of 85% and a design effect of 1.5 to account for the cluster sampling design.

### Sampling methodology

In the first stage of sampling, all the villages in the catchment areas were listed, along with their populations, in a Microsoft Excel worksheet. If the villages were large, they were divided into segments in such a manner that each segment provide at least 50 individuals aged 40 years or older. The study clusters were selected from the list using random numbers generated by Microsoft Excel. For the second stage sampling, 'EPI (Extended Programme of Immunization) Random Walk' method was used to select individuals fulfilling the age criterion. In this method, the study team identified the centre of the cluster by surveying the village with the help of the villagers. After reaching the centre of the village, a random direction was selected by spinning a bottle. The use of this technique for second stage sampling is also reported from various studies [14-16].

### Study area

The population of Prakasam district was estimated at 3.0 million in 2001, with an annual growth rate of 1.08% [17]. About 30% of the population is aged 40 year and older. This district is divided into 56 mandals (administrative blocks below the level of a district). The RAVI study was conducted in the coastal villages spread over an area of 102 km in Prakasam district, Andhra Pradesh. The marine fishing communities live in the hamlets of the big villages located near the sea shore. These communities have a distinct culture, different language for sub ethnic group and are largely dependent upon the sea for their livelihood. Male members are engaged in fishing in the sea and female members sell the fish.

### Ethics approval

The study protocol was approved by the Institutional Review Board of L V Prasad Eye Institute in June 2010. The study was conducted in accordance with the tenets of the Declaration of Helsinki. Data collection was accomplished between July and September 2010.

### Study procedures and personnel

**Figure 1:**
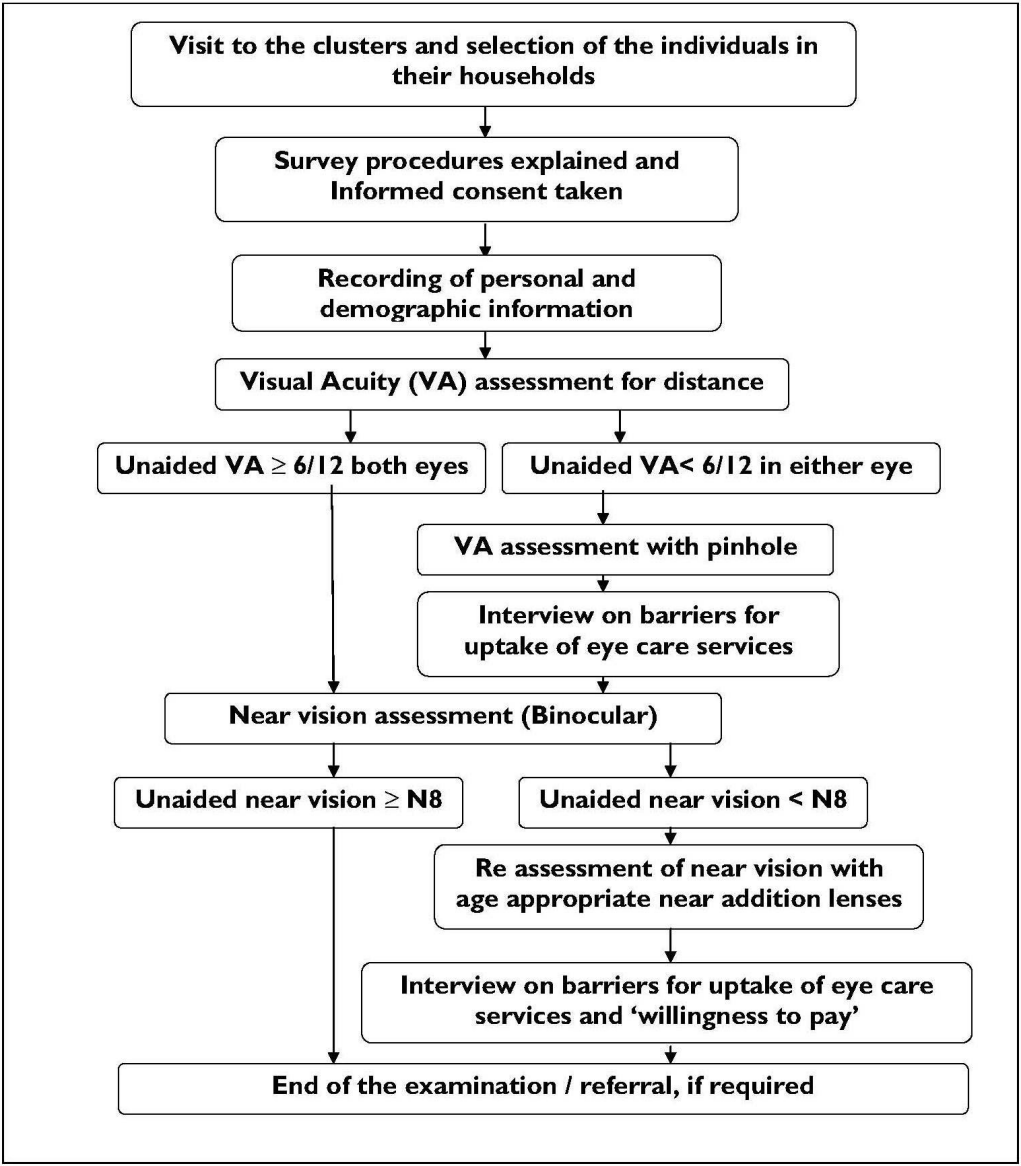
**Flowchart showing the sequence of the study procedures**.

Two teams were used for data collection. Each team consisted of one Vision Technician (trained paramedical ophthalmic personnel) and two community eye health workers. The data was collected in a door-to-door survey in the selected study clusters, and examination was performed in the vicinity of the households. Written informed consent was sought from each subject by the study personnel after explanation of the survey procedures and before starting the examination. The examination protocol is shown in Figure 1.

A two-day rigorous training was imparted, covering topics related to selection of clusters, enumeration methods, clinical examination, coding the form and maintenance of daily records. Inter observer reliability was assessed for visual acuity measurements for distance and near. A minimum kappa static of 0.7 between both the examiners was considered as adequate. Both the eyes of 50 individuals in the clinical setting and 2 clusters in the study area were selected for reliability study.

Distance visual acuity (VA) was measured with a Snellen chart with "E" or English alphabet optotypes. Distance VA was measured outdoors in front of the house. On a bright sunny day, it was measured in the shade. Where this was not possible, the subject was instructed to stand in a way such that the sun was behind the person during VA assessment. Precautions were taken so that there were no reflections from the chart due to sunlight. A plastic rope, 6 meters in length, was used to measure the distance of 6 meters between the individual and the chart. In addition to being easy and fast to use, a rope is more culturally acceptable to people than measuring tape. If the subject was unable to see the 6/60 optotype at the distance of 6 meters, then the distance between the chart and the subject is decreased to 3 meters and VA assessment was done. If the subject failed to identify the largest optotypes, then finger counting was performed.

Unaided (aided, if the subject was using spectacles) and pinhole visual acuity was recorded, if visual acuity < 6/12. VA of both eyes was recorded separately. The aided VA was considering as presenting VA, if a subject was using spectacles and unaided VA was considered as presenting VA, if a subject had no spectacles. Near vision was assessed in all subjects, irrespective of their distance vision, using N notation near vision chart at the customary working distance for each individual (usual range 33-35 cm) binocularly. Unaided (aided, if the subject was using spectacles) was recorded. If an individual failed to read N8, the near vision was re-assessed with age-appropriate near vision spectacles (+1.0 for 40 years and increasing by +0.50 Ds for every five years with maximum of +3.0 Ds for those who are 60 years and above).

A brief personal interview was done to collect demographic information such as details about educational qualification, occupation, spectacle usage, and utilization of eye care services in the past. Torch light examination was performed to assess cataract and anterior segment pathology only if the subject had presenting VA < 6/18 in either eye. The barriers question was asked to all those who had visual impairment due to any cause and/or those who had presbyopia. The question related to willingness to pay was asked to all individuals who had uncorrected refractive errors, presbyopia or cataract. All persons with presenting VA < 6/18 in any eye, or those who needed services were referred to the nearest eye care facility for management. A printed referral letter was provided to the referred subjects.

### Study definitions

Visual Impairment was defined as presenting VA < 6/18 in the better eye. Blindness was defined as presenting VA < 6/60 in the better eye. Moderate visual impairment was defined as presenting VA < 6/18 to 6/60. For comparison with other studies, the WHO definition of blindness defined as presenting visual acuity < 3/60 in the better eye was also used. Uncorrected Refractive Error was defined as presenting VA < 6/18, but improving to 6/18 or better with pinhole. Cataract was defined as opacity of crystalline lens in the pupillary area as seen with torch and causing visual impairment (presenting VA < 6/18 and not improving with pinhole). Posterior segment disease was considered as present, if there was no improvement in VA on using a pinhole, and no obvious media opacity on torch light examination.

In cases where there was more than one cause for visual impairment, the one which was more easily treatable or correctable to achieve a VA ≥ 6/18 was considered as the primary cause of visual impairment. For example, if a patient had an operable cataract and uncorrected refractive error, the cause was marked as refractive errors as it is easier to correct compared to the surgical intervention for cataract as per recommendation of World Health Organization [18].

### Data management

Data management and analysis was conducted using *Epi Info *for Windows software (Division of Public Health Surveillance and Informatics, Centers for Disease Control and Prevention, Atlanta, GA) and SPSS 16.0 (SPSS Inc., Chicago, IL). Point prevalence estimates and 95% confidence intervals were calculated. Strength of association was described using odds ratios (OR) with 95% confidence intervals (CI). The association of visual impairment with demographic variables such as age, gender, education and occupation were assessed using multiple logistic regression analysis.

## Results

Of the 1700 subjects enumerated from 34 clusters, 1560 (91.8%) were available for examination. The mean age of males and females were similar (p = 0.52). There were no significant differences in the proportion of individuals in the population and study cohorts under each age group category. 45.4% of the subjects were male and 84.1% were uneducated. Marine fishing was the main occupation of the majority of the individuals (28.3%). Those who were not available for examination were significantly younger compared to those examined (p < 0.05). More women were available for examination compared to men (p < 0.05).

### Prevalence and Causes of Visual impairment

The visual impairment was present in 466/1560 individuals (prevalence 30% (95% CI, 27.6-32.2)). This included 111/466 (7.1%; 95% CI, 5.8-8.4) individuals with blindness and 355/466 (22.7%; 95% CI, 20.6-24.8) individuals with moderate visual impairment respectively. Using WHO definition, the prevalence of blindness was 2.7% (95% CI, 1.9-3.5).

The unilateral moderate visual impairment was present in 500/1560 (prevalence 32%; 95% CI, 29.8-34.4) individuals and unilateral blindness was present in 260/1560 individuals (prevalence 16.7%; 95% CI, 14.8-18.6).

On applying multiple logistic regression, the visual impairment was significantly higher in subjects in older age group, male gender, those with no education and those who had retired from work (Table 1).

**Table 1:** Categories of visual impairment

Presenting visual acuity in the better eye	n (%)	95% Cl
Normal (> = 6/18)	1094 (70.1)	67.8 - 72.4
Moderate VI (< 6/18 - 6/60)	355 (22.8)	20.7 - 24.9
Blind (< 6/60 to No perception of light)	111 (7.1)	5.8 - 8.4

**Total**	**1560(100.0)**	

Cataract was the leading cause of visual impairment followed by uncorrected refractive error (Table 2). Cataract was the cause of blindness in 92.8% (103 subjects) and moderate visual impairment in 77.2% (274 subjects) of the individuals respectively. Uncorrected refractive errors was second leading cause of moderate visual impairment, where as post cataract surgical complications were the leading cause of blindness (Table 3).

**Table 2:** Visual impairment and its association with demographic variables (Multiple Logistic Regression analysis)

	n (%)	No. with visual impairment (%)	Odds ratio (95% CI)	*P value*
	(n = 1560)	(n = 466)		
**Age group (yrs)**				
40-49	708	55 (7.8)	1.00	
50-59	402	137 (34.1)	5.44 (3.83-7.73)	*0.00*
60-69	292	165 (56.5)	10.67 (7.26-15.67)	*0.00*
70 and above	158	109 (68.9)	11.81 (7.08-19.69)	*0.00*
**Gender**				
Male	709	219 (30.9)	1.00	
Female	851	247 (29.0)	0.64 (0.44-0.93)	*0.02*
**Education level**				
No education	1312	428 (32.6)	1.00	
Primary school	248	38 (15.3)	0.39 (0.25-0.61)	*0.00*
**Occupation**				
Unemployed	110	31 (28.2)	1.00	
Fishing	441	97 (22.0)	0.98 (0.55-1.74)	*0.95*
Selling fish	235	59 (25.1)	1.17 (0.66-2.05)	*0.59*
Daily wage earner	332	53 (15.9)	0.82 (0.46-1.42)	*0.47*
Home duties only	147	51 (34.7)	1.65 (0.90-3.04)	*0.10*
Retired	238	168 (70.6)	2.93 (1.67-5.15)	0.00
Others	57	7 (12.3)	0.54 (0.21-1.40)	0.20

**Table 3:** Causes of visual impairment (n = 466)

Primary Cause	Moderate Visual Impairment (Presenting visual acuity < 6/18 - 6/60 in the better eye) n (%)	Blindness (Presenting visual acuity < 6/60 - No light perception in the better eye) n (%)	Total
Cataract	274 (77.2)	103 (92.8)	377 (80.9)
Uncorrected Refractive Error	76 (21.4)	1 (0.9)	77 (16.5)
Surgery related complications	5 (1.4)	5 (4.5)	10 (2.1)
Others (corneal scar, phthisis bulbi)	0 (0.0)	2 (1.8)	2 (0.4)

**Total**	**355 (100.0)**	**111 (100.0)**	**466 (100.0)**

## Disscusion

The current research demonstrated the application of novel RAVI methodology to determine the prevalence and causes of visual impairment in subjects aged 40 years and above in coastal fishing communities of Prakasam district in South India. This is the first paper reporting on the visual impairment in this marine fishing community from Andhra Pradesh in India.

The current study revealed that the prevalence of blindness and moderate visual impairment was 7%, and 23.0% respectively compared to blindness prevalence of 8% and visual impairment of 16.5% in Rapid Assessment of Avoidable Blindness (RAAB) survey in India [19]. The prevalence of blindness in the present study is similar to that found in RAAB survey in India and higher than the prevalence of 4.6% reported from Nepal despite inclusion of younger age group in the present study compared to ≥ 50 years older individuals in other two studies [20].

In two surveys conducted in Sri Lanka and China among those aged 40 years and older, the prevalence of blindness was 1.1% (95% CI, 0.3-2.0) and 1.9% (95% CI, 1.5-2.3) respectively compared to 2.7% (95% CI, 1.9-3.5) in the current study [21,22]. The best corrected visual acuity was used in Sri Lankan study and presenting visual acuity was used in the study in China and in present study.

Cataract is the leading cause of visual impairment in all the above studies discussed despite the different definitions and age groups included. However there was a difference in proportion of blindness caused due to cataract. This can possibly be attributed to difference in the population demographics in these areas. It is also possible that the social and economic barriers along with availability of services may limit the uptake of cataract surgery in this region. The higher prevalence of cataract in the present study can possibly be attributed to the prolonged effect of sunlight as these people spend a lot of time on sea by virtue of their occupation. A report on fishermen in Hong Kong found higher prevalence of cataract due to exposure to sunlight [23]. However, this study is not designed to provide a definitive answer to study the relationship between sunlight exposure and cataract.

Extrapolating the prevalence of visual impairment to the about 50000 population of fishing community living in the coastal regions of Prakasam district, about 5000 people aged 40 years and older may have visual impairment and majority of them can benefit from eye care services.

In the present study, the participation was higher in older age groups and among women. The men in younger age groups go out early in the day in their boats for fishing and hence not available for examination. Because of the high response rate in this study, results may not have been biased due to this non availability.

One of the inherent drawbacks of this rapid assessment is the possible over-estimation of cataract as posterior segment examination is not performed. It is possible that some of those with media opacities may have glaucoma, diabetic retinopathy and/or other posterior segment diseases like Age Related Macular Degeneration. However, the prevalence of all these causes compared to cataract alone is much lower, and hence the data from this study still holds good for planning eye care services to control avoidable blindness which mainly focus on cataract surgical services.

The overarching goal of eye care service provision is to reduce the burden of visual impairment through evidence-based planning and regular monitoring of services. The methodology used was simple and straight forward, locally available human resources were used for data collection. The data collection was completed in less than 12 weeks. Due to simplified and rapid data collection, the study can be repeated at regular intervals to track the changes in the prevalence and causes of visual impairment over time.

## Conclusion

Cataract and uncorrected refractive errors which are considered to be the easiest causes of avoidable visual impairment are contributing to over 95% of the visual impairment. The data obtained from this marine fishing community through this study can now be used as the baseline to initiate eye care services in this region. The methodology described in this paper can be replicated in different areas and can become a handy tool for the planning and management of eye care services.

## Competing interests

The authors declare that they have no competing interests.

## Authors' contributions

SM conceived the idea and developed the protocol, reviewed the literature and wrote the initial draft of the manuscript. SRM was instrumental in data collection and assisted in writing of this manuscript. GNR reviewed the initial draft of the manuscript and provided the inputs. All the authors approved the final version of the manuscript.

## Pre-publication history

The pre-publication history for this paper can be accessed here:

http://www.biomedcentral.com/1471-2415/11/26/prepub
